# A Rare Case of a Patient Being Alert and Communicative Despite Severe Hypothermia

**DOI:** 10.7759/cureus.56293

**Published:** 2024-03-16

**Authors:** Emile Jeunesse, Patrick O'Malley, Nick Petrus, Chelsea McCoy

**Affiliations:** 1 Emergency Medicine, Edward Via College of Osteopathic Medicine, Spartanburg, USA; 2 Emergency Medicine, Newberry County Memorial Hospital, Newberry, USA; 3 Internal Medicine, Newberry County Memorial Hospital, Newberry, USA

**Keywords:** external rewarming, osborn waves, atrial fibrillation with rapid ventricular response, low-reading thermometer, accidental hypothermia, severe hypothermia

## Abstract

Hypothermia is defined as a significant drop in core body temperature below 35°C (95°F). It is traditionally staged as mild, moderate, severe, and profound at temperatures of 35°C to 32°C (95°F to 89.6°F), 32°C to 28°C (89.6°F to 82.4°F), <28°C (<82.4°F), and <24°C (75.2°F), respectively. It can also be classified into the same stages by clinical presentations. We present a patient that fits into two different stages based on core body temperature and clinical presentation.

A 58-year-old homeless male with a history of seizures and alcohol use presented via emergency medical services after spending the night outside and uncovered with a core body temperature of 25.1°C (77.1°F) via a urinary bladder thermometer, meeting criteria for severe, near profound, hypothermia. However, he was alert and communicating, shivering, with tachycardia, tachypnea, normal oxygen saturation, and elevated blood pressure, suggestive of mild hypothermia clinically. Passive and active external and internal rewarming were utilized to treat, with the removal of wet clothing, forced air patient warming system, warm blankets, and warm lactated ringers given intravenously. He was soon transferred to the intensive care unit and first returned to normothermic levels after approximately 10 hours from presentation. An electrocardiogram was obtained after resolution of shivering and revealed atrial fibrillation without Osborn waves. He remained in the hospital for the following week to treat his atrial fibrillation, hypothermia-induced rhabdomyolysis, and alcohol withdrawal. He was discharged without neurologic deficits and medically stable with appropriate resources.

This case demonstrates a unique presentation of severe hypothermia. To our knowledge, there has not been a reported case of severe hypothermia that does not involve severe central nervous system depression, severe slowing of vitals, and/or comatose status. These clinical symptoms normally begin during moderate hypothermic levels near 32°C (89.6°F), yet our patient presented without any central nervous system depression and with accelerated vitals that are more consistent with mild hypothermia yet had a core temperature of 25.1°C (77.1°F). Treatment was dictated by his core body temperature rather than clinical presentation. Because of this incongruence between symptoms and true severity of disease in hypothermia, we recommend diagnosis and treatment of hypothermia always be confirmed and based on core body temperature via a low-reading thermometer instead of clinical presentation alone.

## Introduction

Hypothermia is an uncommon condition describing a significant drop in core body temperature below 35°C (95°F) that can result from environmental exposure, shock, or other medical conditions and is potentially life-threatening. Accidental hypothermia is defined as an unintentional fall to hypothermic temperatures, and isolated cold exposure is typically the most common cause of accidental hypothermia [1]. Hypothermia is staged traditionally as mild, moderate, and severe, with mild hypothermia core temperatures ranging from 35°C to 32°C (95°F to 89.6°F), moderate core temperatures from 32°C to 28°C (89.6°F to 82.4°F), and severe core temperatures from <28°C (<82.4°F) [2,3]. Additionally, some experts have begun including core temperatures <24°C (75.2°F) or <20°C (68°F) as “profound hypothermia” [4,5]. Primary hypothermia refers to excessive cold environmental exposure, while secondary hypothermia refers to other causes involving impaired thermoregulation, such as shock or other medical conditions [2].

Epidemiology studies for hypothermia reveal that adults aged 30 to 49 years are most commonly affected, men more than women, and adults aged over 65 years are most at risk for death [6]. The annual incidence of hypothermia-related mortality reported by the Centers for Disease Control from 2018 to 2020 was highest in males in rural areas, 0.93 per 100,000 persons, and lowest in females in metropolitan areas, 0.10 per 100,000 persons [7]. Associated risk factors for hypothermia in adults include severe environmental exposure such as wind, snow, or cold water immersion, older age, alcohol and drug ingestion, homelessness, inappropriate clothing or equipment in extreme environments, trauma, surgery and anesthesia-related resuscitation, sepsis, and/or psychiatric conditions predisposing to a higher likelihood of inappropriate weather behavior [6].

Clinical presentation for hypothermia varies among patients and by stage. Patients with mild hypothermia typically demonstrate shivering, tachypnea, tachycardia, ataxia, dysarthria, impaired judgment, and cold diuresis [1]. Cold diuresis is increased urine production secondary to increased core blood flow that is attempted to restore core body temperature [1]. Patients with moderate hypothermia demonstrate progressive bradycardia and decreased cardiac output, hypoventilation, loss of shivering, central nervous system depression, decreased reflexes, decreased renal blood flow, atrial fibrillation or other arrhythmias, and slowing of pupillary constriction and dilation [1]. Patients with severe or profound hypothermia can present with coma, pulmonary edema, oliguria, areflexia, hypotension, bradycardia, ventricular arrhythmias, asystole, and loss of oculocephalic reflexes [1]. Poorer prognosis is more evident when symptoms are not reversed when the patient is rewarmed, such as fixed, dilated pupils, central nervous system depression, and/or apparent rigor mortis.

Initial assessment and treatment are dependent upon accurate determination of core temperature, most often requiring the use of a low-reading thermometer, as most standard thermometers only read to a minimum of 34°C (93.2°F) and therefore are inappropriate for a hypothermic patient [1,5]. After diagnosis and staging of hypothermia, laboratory evaluation is warranted for potential complications and comorbidities, such as lactic acidosis, rhabdomyolysis, bleeding, and/or infection [1]. An electrocardiogram should also be obtained to evaluate for cardiac arrhythmias [1]. Osborn waves, distortion of the earliest phase of membrane repolarization, may also be present and suggestive of hypothermia, but are not pathognomonic and can be found in subarachnoid hemorrhage, brain injury, and other conditions [8,9]. Treatment involves immediate evaluation and support of the airway, breathing, and circulation, prevention of further heat loss, initiation of rewarming according to degree of hypothermia, and treatment of underlying comorbidities and complications [3,5,10].

Rewarming techniques are commonly described as passive external rewarming, active external rewarming, and active internal core rewarming, related to the stage of hypothermia [1]. Passive external rewarming involves removing wet clothing, adding blankets/insulation, and adjusting room temperature to a heated environment of approximately 28°C (82.4°F), with a recommended rate of rewarming between 0.5°C to 2°C (0.9°F to 3.6°F) per hour [1,5,10,11]. Active external rewarming is in addition to passive external rewarming and involves warm blankets, heating pads, warm baths, and forced warm air to the skin, focusing on the trunk before extremities, with a rate of rewarming at least 2°C (3.6°F) per hour [1,3,5]. Active internal core rewarming is in addition to passive and active external rewarming and involves endovascular treatment with warmed isotonic crystalloid, addition of warmed irrigation of the peritoneum or thorax, and, if the patient is in cardiac arrest or instability, extracorporeal life support, with a rate of rewarming from 2°C to 3°C (3.6°F to 5.4°F) or more per hour [1,3,5,12]. Mild hypothermic patients typically receive passive external rewarming and possibly active external rewarming, while moderate hypothermic patients will receive both passive and active external rewarming and possibly active internal core rewarming, and severe hypothermic patients will receive all techniques with the possible addition of more aggressive active internal core rewarming, such as extracorporeal life support, as needed.

## Case presentation

A 58-year-old African American male with a past medical history significant for seizures, medication non-compliance, and alcohol abuse was brought into the emergency department via emergency medical services with concerns for hypothermia. He was reported homeless in a rural population and found uncovered, outside on the porch of an abandoned house, smelling of wood smoke with an unclear source. He had been outside all night with the weather dropping to -2.78°C (27°F). Upon entrance into the emergency department, the primary survey revealed that he was shivering, mumbling coherent words of discomfort and agitation, experiencing spontaneous eye-opening, and would localize to pain, GCS 14. He was extremely cold to the touch with dry skin and some wet clothing, with a patent airway and no signs of trauma. Vitals signs were as follows: a pulse of 110 beats per minute with a regular rhythm, 130/81 mmHg for blood pressure, 20 non-labored respirations per minute, and 100% oxygen saturation. Oral temperatures were unsuccessful; therefore, rectal temperatures were attempted but only read “low”, finally a urinary bladder thermometer was placed, which revealed a core temperature of 25.1°C (77.1°F). The secondary survey identified a generally ill-appearing male but the remainder of his physical exam was unremarkable aside from the primary survey findings.

Initial intervention in the emergency department included removing wet clothing, applying a forced air patient warming system, warm blankets, and a liter of warm lactated ringers via two peripheral intravenous sites given over the first hour to initiate re-warming. Lorazepam 2mg intravenous was also given as the patient was slightly agitated and it was uncertain if he was compliant with his seizure medications. Labs and imaging were obtained: complete blood count, complete metabolic panel, troponin, creatine phosphokinase, prothrombin, partial thromboplastin time, international normalized ratio, lactic acid, arterial blood gas, urinalysis, drug screen, coronavirus 19, influenza, computer tomography of head/brain without contrast, electrocardiogram. The complete blood count was unremarkable other than mild neutropenia which he had documented before, 2.50K/uL. Significant complete metabolic panel findings included: sodium 124mmol/L (reference range: 135-145mmol/L), magnesium 1.5mg/dL (reference range: 1.6-2.1mg/dL), and total creatine kinase 1073IU/L (reference range: 55-170IU/L). He also had a minimal rise in aspartate aminotransferase and alanine transaminase, 76IU/L and 25IU/L (reference ranges: 10-40IU/L and 7-56IU/L), respectively, which were to be expected with prior alcohol use. His lactic acid was significant at 5.98mmol/L (<2mmol/L). Arterial blood gas showed normal carboxyhemoglobin at 1.4% (<2%), but significant pH 7.189 (reference range: 7.35-7.45), partial pressure of carbon dioxide 43mmHg (reference range: 35-45mmHg), and bicarbonate 16.4mmol/L (reference range: 22-29mmol/L). The ethyl alcohol level was significant at 102.00mg/dL (reference range: 0-50mg/dL). Urinalysis and drug screen were negative. Coronavirus 19 and influenza were negative. Head/brain computed tomography was negative for any acute findings. An electrocardiogram was attempted but was unreadable due to artifact from shivering, therefore it was unclear if Osborn waves were present or not in the emergency department. The patient remained stable throughout his time in the emergency department with slowly increasing temperature and was subsequently admitted to the intensive care unit with primary diagnosis of severe hypothermia and secondary diagnoses of rhabdomyolysis, hyponatremia, hypomagnesemia, and alcohol abuse. His core temperature had risen to 29.6°C (85.3°F) upon admission, three hours after arrival at the emergency department.

**Figure 1:**
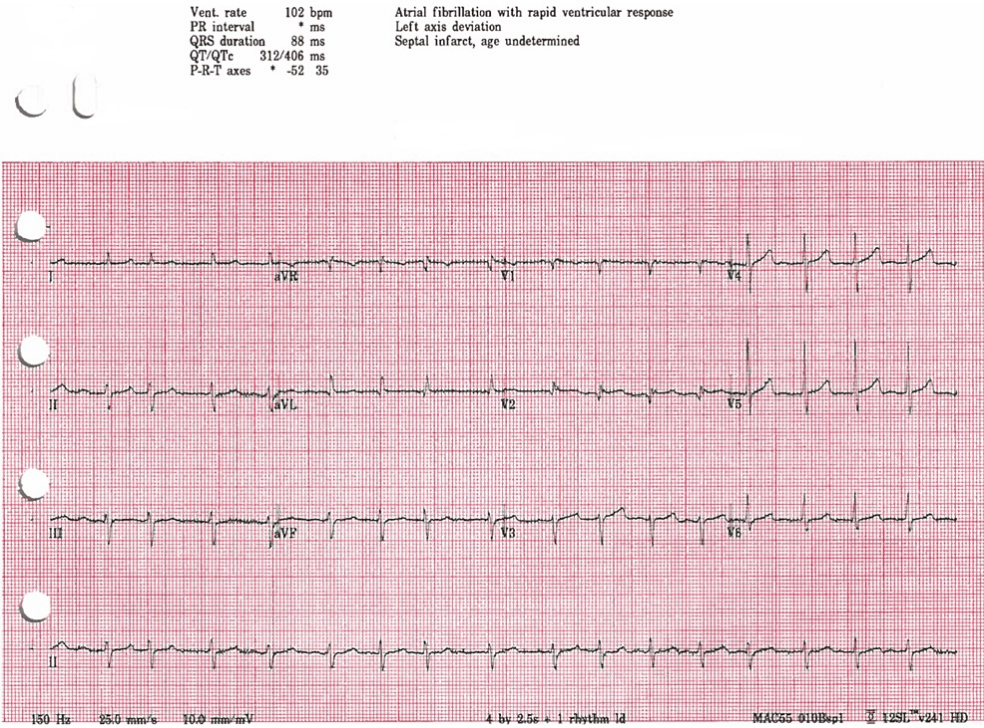
Earliest obtainable electrocardiogram First electrocardiogram obtained with reduced shivering. Atrial fibrillation with rapid ventricular response at 102 beats per minute but no Osborn waves noted.

Upon admission to the intensive care unit, he received continued warming and multivitamins, folic acid 1mg, thiamine 100mg intravenous for potential alcohol withdrawal, and a normal saline bolus followed by 100mls/hr intravenous for rhabdomyolysis. An electrocardiogram was still unattainable because the patient was shivering in the intensive care unit. His core temperature continued to steadily rise and finally reached 36.5°C (97.7°F) approximately 10 hours after arrival at the emergency department. Rewarming procedures were stopped shortly after. His core temperature then remained within normal limits without additional warming for the remainder of his admission. After the patient reached normal temperature limits and was no longer shivering, an electrocardiogram was obtained which revealed atrial fibrillation with rapid ventricular response and no Osborn waves (Figure 1). He was started on heparin 5000 units every 12 hours with the hope of spontaneous conversion while continuing to maintain normal body temperature. Supplemental magnesium and potassium were also given with intravenous fluids to further correct underlying electrolyte imbalances.

On his second day in the intensive care unit, thrombocytopenia of 106k/uL was found (reference range: 150-450k/uL); therefore, heparin was held. Metoprolol 50mg was added for heart rate control since he was still in atrial fibrillation but was asymptomatic. Intravenous fluids were stopped at this time and medications and vitamins were continued orally. Phenytoin 100mg twice a day orally was also started for his seizure disorder. He was moved to the medical floor on the third hospital day, with his temperature remaining stable at 37°C (98.6°F). Lorazepam 2mg intravenous as needed was started secondary to alcohol withdrawal and agitation. On day four, he began working with physical, occupational, and speech therapies to improve generalized weakness and fall risk. On day five, he complained of trouble eating and was found to have an infected right inferior-posterior molar. Thus, amoxicillin/clavulanate 875mg twice a day was begun orally.

**Figure 2:**
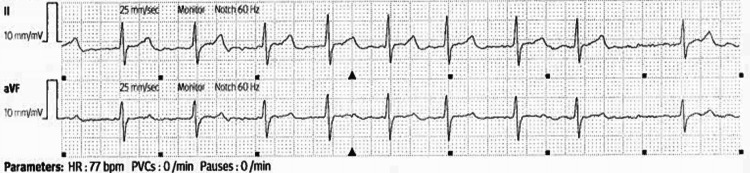
Final electrocardiogram strip before discharge Electrocardiogram strip demonstrating atrial fibrillation at a controlled rate of 77 beats per minute.

He was finally discharged on day seven, being able to walk 250 feet unsupported with physical therapy. He remained in atrial fibrillation on discharge but at a controlled rate of 77 bpm (Figure 2). He was discharged with five more days of antibiotic prescription for the molar infection and several months' worth of metoprolol tartrate 50mg twice a day and phenytoin 100mg twice a day for atrial fibrillation and seizure control, respectively. Before he was discharged, he had spoken with case management and was provided resources for housing, food, and alcohol cessation. He was advised to follow up with a dentist and a primary care provider, with resources provided. His core temperature upon discharge was 37.1°C (98.8°F).

## Discussion

The lowest recorded core temperatures for accidental hypothermia among individuals who have been successfully resuscitated were 13.7°C (56.7°F) in a 29-year-old female and 11.8°C (53.2°F) in a two-year-old male [13,14]. The lowest recorded core temperatures in induced reversible hypothermia with successful resuscitation were 9°C (48.2°F) and 4.2°C (39.6°F) [14,15]. There are numerous additional case reports of patients with severe hypothermia, <28°C (<82.4°F), and profound hypothermia, <24°C (<75.2°F), all of whom successfully recovered, many with full neurologic recovery [2,6,16-18]. These patients all suffered various but significant central nervous system depression and slowing of vital signs during hypothermia, with many moving into comatose status. To our knowledge, a severe hypothermia case without this common presentation has not been reported. However, as detailed above, our patient suffered vital signs and symptoms that would be classified as mild hypothermia, while his core temperature would be classified as severe, if not nearly profound, hypothermia at 25.1°C (77.1°F).

One probable reason for this discrepancy is the use of a urinary bladder thermometer. Pulmonary artery thermometers are considered the gold standard for measuring core body temperature accurately and without lagging behind the body’s true core temperature [19]. However, esophageal and urinary bladder thermometers are safer and more frequently utilized, especially in emergency settings [19,20]. A review of various studies and thermometer comparisons has shown that esophageal thermometers are comparable to pulmonary artery thermometers, with more accuracy and less lag time than urinary bladder thermometers, but both are effective for determining core body temperature in emergency settings [19,20]. One study demonstrated that urinary bladder thermometers lagged behind esophageal thermometer readings by approximately one hour +/- 30 minutes, both when patients were being cooled and rewarmed [20]. Therefore, it could be possible that the urinary bladder temperature in our patient did not reflect a true core body temperature, his true core body temperature effectively being warmer than the urinary bladder thermometer’s reading. However, this same study also reported those differences were approximately 0.4°C +/- 0.3°C (0.72°F +/- 0.54°F) at most, and that esophageal readings were always lower than urinary bladder thermometer readings, even during rewarming phase [20]. Thus, it is also likely that the urinary bladder thermometer in our patient may have possibly reported a warmer temperature than his true core temperature. With only mild hypothermia symptoms, our particular patient was not an appropriate patient for an esophageal thermometer to compare, but the limitation is important to note and probable.

Our patient demonstrates a difficult dilemma: diagnosis of mild hypothermia based on clinical presentation or diagnosis of severe, near profound, hypothermia based on core body temperature? It is unfortunate that an electrocardiogram was unable to be obtained early on due to shivering, as the presence of an apparent arrhythmia could have further refined a clinical diagnosis. It is difficult to say with certainty whether the atrial fibrillation discovered later in his hospital stay was present upon initial presentation to the emergency department or only began after rewarming. Ultimately, he was diagnosed and treated with severe hypothermia because emergency medicine addresses the most severe possibility first and attempts to prevent further complications from occurring; this also held in the best interest of the patient. Alternatively, it is also likely a patient could present with mild hypothermia via core body temperature and with severe hypothermia presentation clinically. In this type of setting, it is more likely that the clinical presentation would be related to an alternative etiology than true hypothermia, and a detailed medical work-up would be pursued to rule in/out other etiologies. Therefore, from our perspective, it is more useful to stage hypothermia based on core body temperature rather than clinical symptoms. As a result, having quality low-reading thermometers, such as urinary bladder and esophageal thermometers, available in emergency settings is crucial for appropriate medical intervention for hypothermia.

Additionally, our case adds to the growing body of literature on hypothermia. Our patient was congruent with known risk factors for accidental hypothermia: excessive environmental exposure, homelessness, and alcohol use [6]. He suffered rhabdomyolysis consistent with accidental hypothermia complications. While our patient did not have Osborn waves, he did present with atrial fibrillation upon rewarming, consistent with reports of hypothermia-associated arrhythmias [1,8,9]. Treatments for severe hypothermia, such as extracorporeal warming measures with warm, intravenous crystalloid solutions, were beneficial and showed a warming rate of 2°C to 3°C (3.6°F to 5.4°F) for our patient, consistent with recommendations and guidelines [1,3,5,12].

## Conclusions

This case involves a unique presentation of a patient with severe, near profound, hypothermia at 25.1°C (77.1°F) yet demonstrating clinical findings of alertness and vital signs consistent with mild hypothermia. While successful resuscitation of patients at much lower temperatures is well reported, there is no report of a patient with such a low core temperature with so few clinical symptoms. Such a case adds to the growing body of literature on hypothermia. In conclusion, because of this case’s incongruence between symptoms and true severity of disease in hypothermia, we recommend that diagnosis and treatment of hypothermia always be confirmed and based on core body temperature via a low-reading thermometer instead of clinical presentation alone.

## References

[1] Paal P, Brugger H, Strapazzon G (2018). Accidental hypothermia. Handb Clin Neurol.

[2] Rasmussen JM, Cogbill TH, Borgert AJ (2022). Epidemiology, management, and outcomes of accidental hypothermia: a multicenter study of regional care. Am Surg.

[3] Lott C, Truhlář A, Alfonzo A (2021). European Resuscitation Council Guidelines 2021: cardiac arrest in special circumstances. Resuscitation.

[4] Durrer B, Brugger H, Syme D (2003). The medical on-site treatment of hypothermia: ICAR-MEDCOM recommendation. High Alt Med Biol.

[5] Paal P, Pasquier M, Darocha T (2022). Accidental hypothermia: 2021 update. Int J Environ Res Public Health.

[6] Petrone P, Asensio JA, Marini CP (2014). Management of accidental hypothermia and cold injury. Curr Probl Surg.

[7] (2022). Quickstats: death rates attributed to excessive cold or hypothermia, by urbanization level and sex — national vital statistics system, 2018-2020. MMWR Morb Mortal Wkly Rep.

[8] Alsafwah S (2001). Electrocardiographic changes in hypothermia. Heart Lung.

[9] Graham CA, McNaughton GW, Wyatt JP (2001). The electrocardiogram in hypothermia. Wilderness Environ Med.

[10] Dow J, Giesbrecht GG, Danzl DF (2019). Wilderness Medical Society Clinical Practice Guidelines for the out-of-hospital evaluation and treatment of accidental hypothermia: 2019 update. Wilderness Environ Med.

[11] Mydske S, Thomassen Ø (2020). Is prehospital use of active external warming dangerous for patients with accidental hypothermia: a systematic review. Scand J Trauma Resusc Emerg Med.

[12] Austin MA, Maynes EJ, O'Malley TJ (2020). Outcomes of extracorporeal life support use in accidental hypothermia: a systematic review. Ann Thorac Surg.

[13] Gilbert M, Busund R, Skagseth A, Nilsen PA, Solbø JP (2000). Resuscitation from accidental hypothermia of 13.7 degrees c with circulatory arrest. Lancet Lond Engl.

[14] Zafren K, Paal P, Brugger H, Lechner R (2020). Induced hypothermia to 4.2°C with neurologically intact survival: a forgotten case series. Wilderness Environ Med.

[15] NI SA, LE FJ (1958). Profound hypothermia in man; report of a case. Ann Surg.

[16] Kiekkas P, Fligou F, Igoumenidis M, Stefanopoulos N, Konstantinou E, Karamouzos V, Aretha D (2018). Inadvertent hypothermia and mortality in critically ill adults: systematic review and meta-analysis. Aust Crit Care.

[17] Pasquier M, Zurron N, Weith B, Turini P, Dami F, Carron PN, Paal P (2014). Deep accidental hypothermia with core temperature below 24°c presenting with vital signs. High Alt Med Biol.

[18] Ko CS, Alex J, Jeffries S, Parmar JM (2002). Dead? Or just cold: profoundly hypothermic patient with no signs of life. Emerg Med J.

[19] Hymczak H, Gołąb A, Mendrala K (2021). Core temperature measurement-principles of correct measurement, problems, and complications. Int J Environ Res Public Health.

[20] Umińska JM, Buszko K, Ratajczak J (2020). Comparison of temperature measurements in esophagus and urinary bladder in comatose patients after cardiac arrest undergoing mild therapeutic hypothermia. Cardiol J.

